# History and evolution of cytogenetics

**DOI:** 10.1186/s13039-015-0125-8

**Published:** 2015-03-20

**Authors:** Malcolm A Ferguson-Smith

**Affiliations:** Department of Veterinary Medicine, University of Cambridge, Madingley Road, Cambridge, CB3 0ES UK

## Abstract

The events that have led to the development of cytogenetics as a specialty within the life sciences are described, with special attention to the early history of human cytogenetics. Improvements in the resolution of chromosome analysis has followed closely the introduction of innovative technology. The review provides a brief account of the structure of somatic and meiotic chromosomes, stressing the high conservation of structure in plants and animals, with emphasis on aspects that require further research. The future of molecular cytogenetics is likely to depend on a better knowledge of chromosome structure and function.

## Introduction

Our specialty was pioneered by scientists who developed the compound microscope to study the cellular organisation of the living world. While comparative anatomists had known for centuries that all animals share physical features that suggest a common structure among creatures both living and revealed in fossils, the cytologists of the 19^th^ century found that this concept extended to a cellular organisation present in all plants and animals. Variations in morphology within species, and to a greater extent between species, led Linnaeus and other taxonomists to classify all organisms in terms of genealogies with species, families and orders depending on their similarities, starting with individuals capable of reproduction that defined a species. The stage was set for ideas about the transmutability of species, the heritability of physical traits and Darwin’s theory of the origin of species [1].

The mechanisms of transmission of both discontinuous and continuous characteristics across the generations were unknown before Mendel’s laws were explained at the turn of the 20^th^ Century by the behavior of chromosomes in germ cells [2,3]. Stains used by pathologists to identify bacteria also served to identify chromosomes. Proof of the chromosomal theory of inheritance was a decisive event in biology that turned cytologists into cytogeneticists. Morgan, Sturtevant, Bridges and Muller constructed the first genetic linkage maps from recombination studies in crosses made in the fruit fly and from cytological preparations of its polytene salivary gland chromosomes [4-6]. Cyril Darlington pioneered plant cytogenetics in 1920–30 and made important advances in our understanding of mechanisms of chiasma formation and the behavior of sex chromosomes in meiosis [7]. These studies reaffirmed that chromosome structure and behavior in somatic and germ cell divisions were common to all plants and animals.

In 1944 it was realized that genetic transformation in bacteria was due to DNA and not protein and that DNA was the molecule responsible for heredity in genes and chromosomes [8]. The molecular structure of DNA became a key question. Chargaff showed in 1950 that, in DNA, the amount of adenine is equal to the amount of thymine and that the amount of guanine is equal to the amount of cytosine [9]. This was the important clue to the structure of the DNA double helix modeled by Watson and Crick in 1953, and based on the X-ray diffraction studies of Rosalind Franklin [10]. Some eight years later it was discovered that triplets of the base pairs specified each amino acid in the polypeptide chain of each protein [11,12]. The sequence of base pairs in DNA/RNA is thus the universal genetic code in all forms of life that descended from a common progenitor 4.5 billion years ago. Phylogenomic studies using chromosome painting confirms the high conservation of DNA between even distantly related species [13].

### Chromosome structure

Since the genetic code was deciphered much has been learnt about the chromosome structure shared by all organisms from yeast to human. Much more remains to be discovered. One of the purposes of this review is to encourage research into chromosome structure as this could help advance molecular cytogenetics. The following is a brief summary of the author’s view of current knowledge, emphasizing areas that need further study.

We now recognize that, following DNA replication, the metaphase chromosome consists of two chromatids held together by a centromere and by cohesin. Each chromatid is a single molecule of DNA attached to protein matrix fibres that forms its scaffold or axial filament [14]. Over 200 different proteins are associated with chromatin [15]. The sites of DNA attachment to the scaffold have not been sequenced although repetitive elements are said to be involved. The DNA molecule between matrix attachment sites extends out from the scaffold in a series of loops of chromatin fibres of varying length, the largest loops tending to aggregate into chromomeres [16]. The chromatin fibres vary in compaction from a nucleosome-free molecule to an 11 nanomere fibre in which the DNA is wrapped round an octomer of histones to form a nucleosome. Linker histones provide further compaction between nucleosomes and this leads to a 30 nm fibre typical of the chromatin loops which radiate from the scaffold [17].

**Figure 1:**
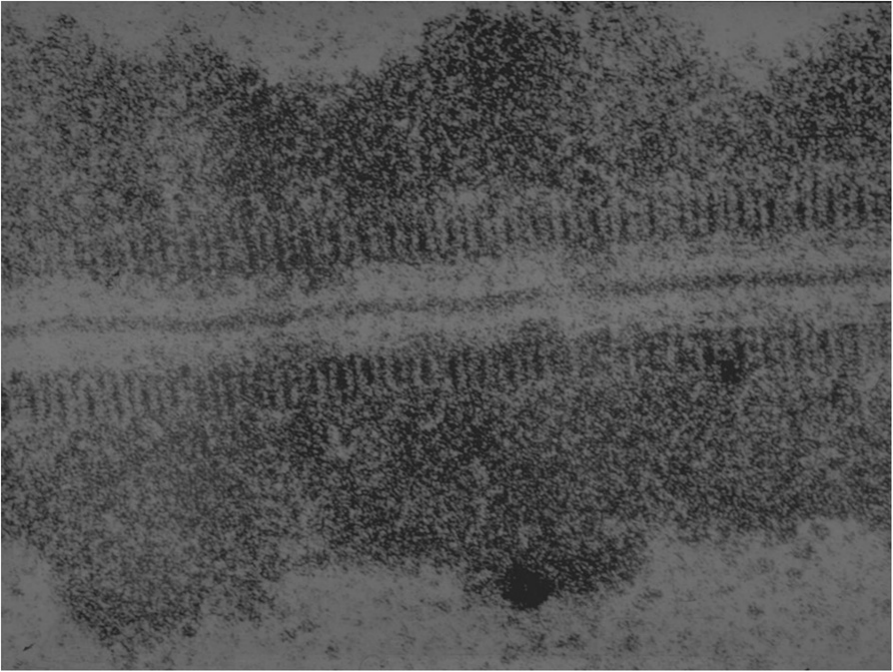
**Electron micrograph of part of a synaptinemal complex in a pachytene bivalent from** ***Neottiella rutilans*** **(from Westergaard and von Wettstein** [20]**).**

During gametogenesis parental homologous chromosomes, each consisting of two chromatids, pair together during the long prophase of the first meiotic division and form chromosomal bivalents. Here again, the two DNA molecules of each parental chromosome are attached to protein matrix fibres that now form the axial filaments of the two lateral elements of the synaptinemal complex (SC, Figure 1) [18]. The process of synapsis that leads to the SC, involves the repair of double-stranded breaks (DSBs) that occur in short chromatin loops which emerge from each lateral element and meet together to form the central element of the SC. Repair of the many DSBs at this site generates a few crossovers but the majority of repaired DSBs result in non-crossovers [19]. It is not known what determines the great preponderance of non-crossovers. The repair of DSBs is associated with detectable DNA synthesis in early pachytene. As the SC disassembles and disappears at late pachytene and early diakinesis, the few DSBs that result in crossovers appear as chiasmata.

Electron microscopy reveals that the SC has a similar structure in all plant and animal species that produce germ cells [20,21]. The lateral elements of the SC are formed by the axial filaments and those chromomeres and chromatin loops that do not seem to take part in synapsis. The regular transverse banding pattern across each lateral element seen in some images (Figure 1) is unexplained. Synapsis may be confined to the short chromatin fibres that arise from each point of attachment to the axial filament and which cross between the two lateral elements and meet in the central element. It is presumed that DSBs repair at complementary sequences in loops from opposite lateral elements. The approximate 0.15 micron distance between the two lateral elements of the SC seems remarkably constant in all species suggesting that short fibres of specific sequence are required for synapsis. It is postulated elsewhere that a special class of chromosome-specific non-coding DNA that does not interrupt essential coding sequences is necessary for synapsis at these sites [22]. (The hypothesis derives from chromosome painting studies that suggest that such a class of functional non-coding DNA is responsible for the extensive hybridization of whole chromosome-specific DNA to chromosomes. This class of DNA may have evolved to prevent non-homologous recombination during meiosis).

**Figure 2:**
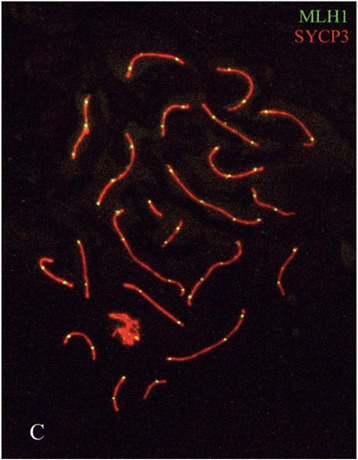
**Pachytene stage in a human spermatocyte in which the synaptinemal complexes are revealed by red immunofluorescence using antibodies to the protein SYCP3.** Recombination nodes are indicated by yellow fluorescence with antibodies to MLH1 (from Sciurano et al. [23]).

Many proteins assist in the pairing of chromosomes during meiotic prophase and the formation of SCs [15,23]. Most have been isolated from testis by chromatography but others have been extracted from organisms such as yeast. Antibodies to these proteins are used to determine their location in chromosome preparations by immunofluorescence (Figure 2). Most revealing are those proteins like SYCP3 that label the SC throughout meiotic prophase, and those like MLH1 that locate recombination nodes where chiasmata are formed [23,24]. Counts of recombination nodes at pachytene correspond to numbers of chiasmata at diakinesis. RAD51 is a protein present during the early repair of DSBs. γH2AX and BRACA1 are found at regions of asynapsis, for example indicating meiotic sex chromosome inactivation at the non-pairing parts of the mammalian XY bivalent [25]. (Transcriptional silencing may occur also at the asynaptic sites in autosomal translocations). These are examples of proteins that have helped to reveal the progress of synapsis and meiotic recombination. However, over 80% of chromosomal proteins are structural proteins, including core and linker histones, topoisomerase II, cohesin and the eight subunits of condensin I and II. Chromosome fibrous proteins are also another important class and these include tubulin, β-actin and vimentin [15]. It is not clear how the protein scaffold replicates during the cell cycle. The nature and number of proteins that make up the matrix fibres of the axial filaments are also unknown.

The structural and fibrous proteins are components of somatic as well as meiotic chromosomes. Scanning electron microscopy reveals instances of crosslinking of chromatin fibres between the chromatids of somatic metaphase chromosomes and this may be the basis of sister chromatid exchange demonstrated by bromodeoxyuridine labeling [26]. Somatic recombination may involve a similar mechanism and, if so, is likely to be mediated by the same meiotic proteins that are associated with DSBs. Indeed, chiasmata have been observed occasionally between homologues in somatic metaphases [27] and illegitimate recombination between homologous regions on non-homologous chromosomes is one of the mechanisms that produce chromosome rearrangements [28].

At interphase the de-condensed chromosome unravels its chromomeres and forms a loose domain of extended chromatin loops that are still attached to the chromosome scaffold. Chromosome painting with whole chromosome-specific DNA probes reveals these domains as distinct chromatin territories equivalent to the number of chromosomes. The observation of six chromatin territories, corresponding to a diploid number of six, that fill the interphase nucleus of the Indian muntjac is a dramatic early example observed in 1994 [29] and illustrated later [30]. After DNA replication the chromatin loops are gathered towards their attachment sites and, as prophase advances, the chromomeres reform so that at metaphase the chromatids once more become cylindrical. In some cells, for example in spermatogonial metaphases, the chromatids adhere closely and the chromosome assumes the shape of a coiled spring. Darlington found that heat treatment of his chromosome preparations exaggerated the major spirals in *Tradescantia* and *Fritillaria* and he believed that chromosome condensation involved a series of minor and major spirals [7]. Just as molecular bonds account for the helical shape of DNA, the three dimensional structure of associated proteins may occasionally induce major spirals in metaphase chromosomes. A better understanding of the structure and role of these proteins may require X-ray crystallography and techniques such as chromatin conformation capture [16].

In the 1970s various chromosome banding techniques were introduced (see below) and these show that G- and Q-bands are associated with A-T rich regions and repetitive DNA, while the regions between bands are associated with G-C rich coding regions including genes. The A-T rich regions also correlate with the chromomere patterns observed in pachytene bivalents [31]. Thus the long chromatin fibres that compact into large chromomeres tend to be A-T rich, have more DNA repeats and are less likely to be involved in synapsis than G-C rich regions.

### The emergence of human cytogenetics

Throughout the first half of the 20^th^ century genetic studies were mostly confined to plant and animal species rather than to humans. It was more productive to make crosses in fruit flies and mice because of the larger number of progeny that could be observed over several generations. However, a number of human pedigrees were collected and characterised [32] and inborn errors were analysed by biochemistry [33]. But these were comparatively rare events and human genetics became more concerned with biometrical genetics and gene frequencies in populations and their mathematical analysis [34]; this led to the useful development of statistics but the neglect of human biological investigations. It was also difficult to study human chromosomes from tissue sections. Crude estimates of the number of chromosomes in human cells at the end of the 19^th^ century gave counts of 16–38 with most in favour of 24 [35-37]. More realistic counts of 47 in testis and 48 in ovary, with a single X sex chromosome in males and two Xs in females, were reported by de Winiwarter in 1912 [38]. In 1921 Painter found the Y chromosome that had been missed earlier and concluded that 48 was the number in both sexes despite noting in 1923 that the best cells showed only 46 [39]. Over the next 33 years at least 40 studies confirmed the diploid number of 48 [40] until Tjio and Levan in 1956 showed that the correct number was 46 [41]. The correction was achieved by using cell cultures and colchicine to accumulate mitoses. The mitotic cells were treated with hypotonic solution to disperse the chromosomes, and then were squashed between the slide and coverslip to spread the chromosomes in one optical plane. (The action of hypotonic treatment was discovered by Hsu, Makino and Hughes independently in 1952 [42-44], all of whom failed to count the correct number of chromosomes). The result of 46 was confirmed immediately by Ford and Hamerton [45] in human meiotic chromosomes.

The first discovery of a human chromosome aberration was made by Marthe Gautier and colleagues from Paris in May 1958. They found an extra small chromosome in fibroblast cultures from several children with Down syndrome [46]. This was announced in 1958 and reported in January 1959. At the same time in Britain sex chromosome abnormalities had been found in the Turner and Klinefelter syndromes, although these were reported several months later. The work on sex chromosome aneuploidy was prompted by the paradoxical nuclear sex chromatin findings previously interpreted as indicating male and female sex reversal respectively. However, patients with the Turner syndrome proved to have a single X and no Y [47], while the Klinefelter patients had an XXY sex chromosome complement [48]. The result in Klinefelter syndrome confirmed an earlier observation of the Y in XY sex bivalents in spermatocytes from a lone fertile tubule in a sex chromatin-positive case [49] These findings provided the first evidence that in mammals sex was determined by a testis-determining factor on the Y chromosome (reviewed in [50]). There was widespread surprise that such gross genetic abnormalities could be viable in humans, and a search was made for other examples. Thus the following year two syndromes due to different extra autosomes, namely trisomies 13 and 18, were reported [51,52]. Like trisomy 21 in Down syndrome, these conditions were recognized by their distinctive patterns of dysmorphology and severe handicap. In all these cases chromosome analysis was made from bone marrow [53] or fibroblast cultures. Nonetheless, the technology was sufficient for a small deletion to be detected in the long arm of chromosome 22 (the Philadelphia chromosome) in the leukaemic cells of patients with chronic myeloid leukaemia [54]. This was the first somatic chromosome aberration to be discovered in a cancer cell, and provided strong support for Boveri’s prediction in 1914 that cancer was caused by chromosomal changes [55].

Chromosome analysis became much easier later in 1960 when lymphocyte cultures were introduced, made from small samples of peripheral blood stimulated by phytohaemaglutinin [56]. Air-dried preparations replaced the squash technique and produced chromosomes of higher resolution. Immediately the technology became simple for all pathology laboratories, and the era of clinical cytogenetics was born. Chromosomal syndromes due to gross deletions, duplications and translocations were soon reported. As some were familial, or associated with a risk of recurrence, it was important to advise parents about these risks and the need for genetic counselling increased. The history of these early events in diagnostic cytogenetics is detailed in many reviews [57-59] and in the monograph “The Beginnings of Human Cytogenetics” by Harper [60]. The latter provides interesting references on early cytogenetics in Russia where hypotonic treatment of mitotic cells was introduced in 1934 [61], 18 years before its use in the West, and where autolysis of red cells was noted to stimulate mitoses in lymphocytes in 1935 [62]. Failures in communication between East and West and the purge of geneticists by Stalin at the time had clearly delayed progress in human cytogenetics for many years. The role of cytogenetics in gene mapping and in the human genome project [63], and its role in the evolution of medical genetics [64] have been discussed extensively elsewhere. The present historical review concentrates on the part played by technical innovation in the development of our specialty.

In previous sections of this article emphasis has been made of several technical milestones in the progress of human cytogenetics. Cell cultures, colchicine and hypotonic treatment led to the correction of the human chromosome number, and lymphocyte cultures to the widespread use of diagnostic cytogenetics and the discovery of many chromosomal syndromes. While the techniques used in the 1960s were sufficient to demonstrate the sex chromosome and autosomal aneuploidies, the Philadelphia chromosome and some of the gross structural aberrations, such as translocation Down syndrome [65], there was a need for better methods of chromosome identification. Only the three largest autosomes, the Y chromosome, chromosomes 16–18 could be recognized with certainty at that time. As prenatal diagnosis was being introduced in 1969 for older mothers, and for young mothers with translocations, improved reliability was essential [66]. Chromosome-specific patterns of DNA replication gave some improvement but were impractical for prenatal diagnosis [67]. Secondary constrictions in chromosomes 9, 11, 16 and 17 were helpful forerunners of chromosome bands [68] but had little application.

The introduction of chromosome banding in 1969–70 has been one of the most important innovations in cytogenetics. The discovery was first made in *Vicia faba* by the group of Caspersson and Zech with quinacrine that intercalated into DNA producing dark and light Q-bands visible by UV microscopy along each chromosome [69]. Lore Zech discovered in the following year that all human chromosomes could be identified from one another by Q-banding [70]. Her pioneering work on Q-banding and its application to the recognition of chromosome aberrations in leukaemia and lymphomas are not sufficiently acknowledged [71]. Meanwhile, Pardue and Gall had demonstrated that isotopic labelled mouse satellite DNA (obtained by ultracentrifugation) could be hybridised *in situ* to the centromeres of denatured mouse chromosomes [72]. The radioactive signals were detected by autoradiography and it was noted that the sites of hybridization on the denatured chromosomes were selectively stained by Giemsa, producing what are now known as C-bands [73]. Various modifications of the denaturing process with alkali, heat or proteolytic enzymes produced alternate light and dark Giemsa bands (G-bands) along the chromosomes; these correspond directly to Q-bands. Trypsin G-banding [74] identifies each human chromosome unambiguously and has been widely adopted in diagnostic cytogenetics to detect aberrations previously invisible. Banding methods have contributed to the precision of gene mapping and cancer cytogenetics, for example in the discovery by Zech and Rowley that the Philadelphia chromosome is an unbalanced 9;22 translocation [71,75]. Many cancers are now thought to arise in stem cells which have undergone massive chromosomal rearrangements due to a single chromothripsis event [76] and banding is essential for their analysis.

**Figure 3:**
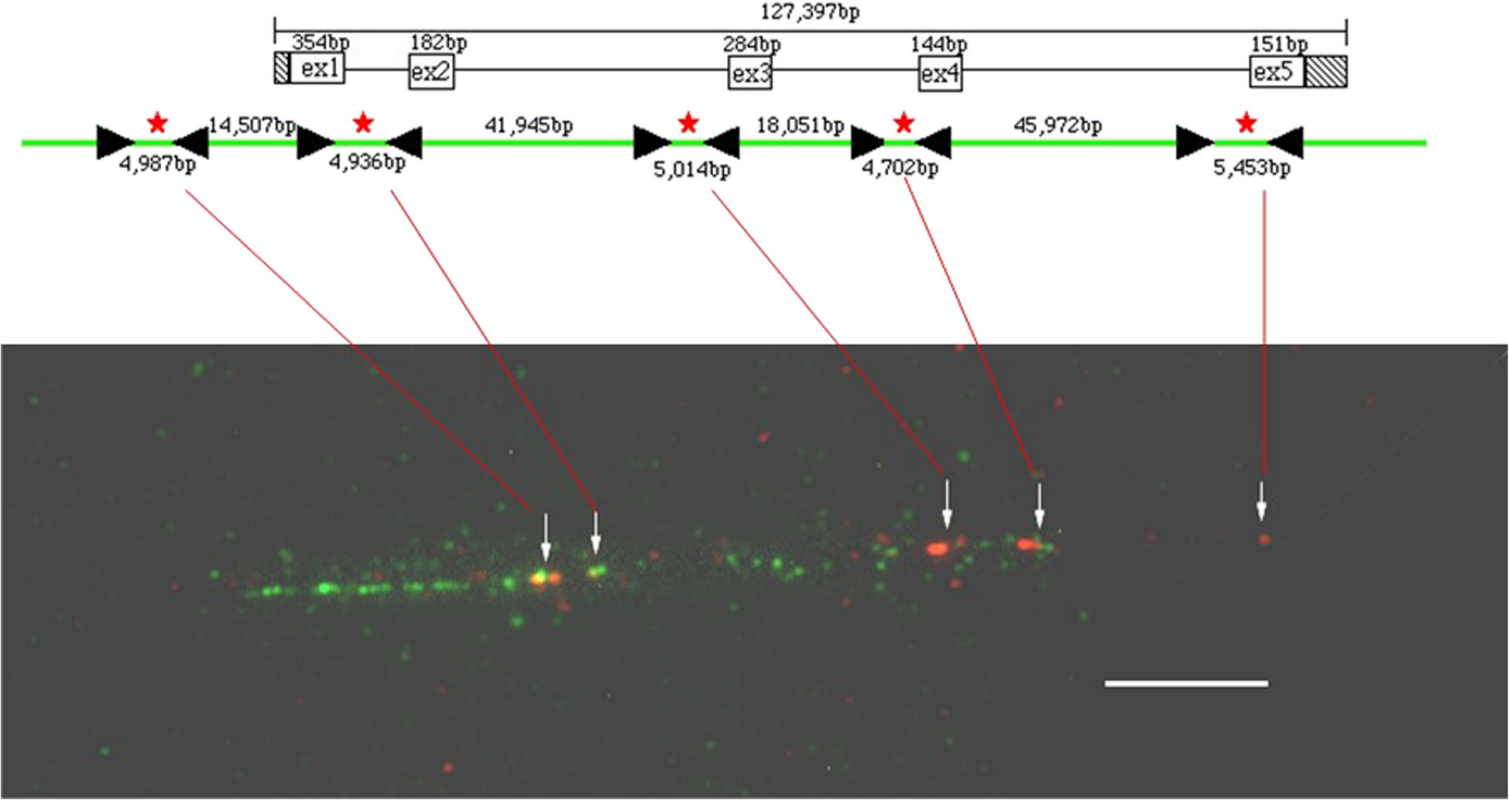
**DNA fibre-FISH showing exon-specific cosmid probes hybridised to the five exons of the DMRT1 gene.** The order of exons is confirmed by the DMRT1 sequence from the human genome database in which distances are indicated by numbers of base pairs. (Unpublished image courtesy of Dr Fumio Kasai).

As mentioned above, Pardue and Gall were the first to use *in situ* hybridization for mapping DNA sequences to chromosomes. The method was used in 1972 to map the ribosomal genes to the short arms of the human acrocentrics [77] but was not successful for single copy genes until recombinant DNA techniques led to the cloning of DNA fragments in phage or plasmid vectors; this allowed the production of sufficient amounts of gene probes to yield detectable signals by autoradiography. In 1981 the genes for the human globins, insulin and kappa immunoglobulin light chains were assigned to chromosomes 16p, 11p, and 2p respectively [78-80]. Because labelling with radioisotopes was time-consuming and impractical, it was soon replaced by fluorescence-labelling and UV microscopy. Fluorescence *in situ* hybridization (FISH) has become the standard method for gene mapping [81,82]. The use of several probes emitting different colours under UV permitted the ordering of gene loci along the chromosome; more closely-linked genes could often be ordered by FISH on the extended interphase chromosome [83]. Further resolution can be achieved by DNA fibre FISH in which chromatin fibres are decondensed by histone depletion and released from their protein scaffold, spread out and fixed on slides [84]. DNA probes can then be hybridized to these greatly extended fibres, so that sequences less than 5 microns apart may be separated. Exons of a single gene have been localised on the same fibre and intronic distances determined and matched to the equivalent sequence in the genome database (Figure 3). The resolution is remarkable and demonstrates that the location and order of genes on a chromosome can be made with higher precision by FISH than by the classical methods of genetic linkage based on recombination data from pedigree analysis.

The above account so far has discussed cytogenetic techniques that are based on conventional or electron microscopy. Flow cytometry is another valuable approach that examines chromosomes in fluid suspension. The dual laser fluorescence-activated cell sorter (FACS) was designed for the analysis of cells stained by immunofluorescence, but has been adapted for measuring and sorting chromosomes on the basis of their size and base-pair ratio [85-87]. The aim is to collect enough of each constituent chromosome in the karyotype so that chromosome–specific DNA can be amplified and labelled by PCR from the collected material. The specificity of the amplified DNA probe is verified by hybridisation to denatured metaphases and this is shown by the probe “painting” its complementary sequences on the correct chromosome. The “paint” probes have obvious application in the identification of chromosome aberrations. In the M-FISH method [88] five fluorochromes are used in different colour combinations to label each chromosome with a unique combination detectable by digital microscopy, so that all chromosomes can be identified in one hybridization and interchromosomal aberrations identified. In reverse painting [89] structurally abnormal chromosomes are isolated by FACS (or by microdissection) and their DNA hybridized to normal metaphases, thus revealing the origin of the chromosomes involved in the aberration. PCR amplification of microdissected chromosomes and parts of chromosomes are used for high resolution colour banding [90,91] and this can be especially valuable for the detection of intra-chromosomal aberrations. Among the practical applications of FISH mention should be made of gene-specific probes used in identifying microdeletion syndromes [27] and centromeric and cosmid-contig probes used extensively in the rapid prenatal diagnosis of aneuploidy in interphase amniotic fluid and other cells [92-94].

Chromosome painting has played an important role in basic research on gene interactions, including regulation, in the interchromatin compartment between chromosome territories in the interphase nucleus [95]. Specific interactions between DNA segments on different chromosome domains can be investigated by such techniques as chromosome conformation capture [16].

Cross-species reciprocal chromosome painting has been most productive in phylogenetic studies in determining the relationships between species and in predicting ancestral karyotypes [96]. The method is most informative for species within but not between placental mammals, monotremes, marsupials and birds. Surprisingly, cross-species painting between birds and reptiles reveals a high degree of conservation despite over 300 Myrs divergence [13]. This approach has applications also in diagnostic cytogenetics in that M-FISH probes made using chromosome-specific DNA from flow-sorted gibbon chromosomes (which are highly rearranged) produce colour bands on human chromosomes useful for aberration detection [96]. An added bonus is that repetitive DNA is sufficiently diverged in gibbons so that background signals are less on human chromosomes than with human paint probes.

It has been shown that chromosome sorting by FACS can be one of the most accurate methods for determining a species genome size and for estimating GC content of individual chromosomes. The method involves sorting a suspension of chromosomes from the test species in a mixture containing a suspension of chromosomes from the control species, such as human, containing several non-heteromorphic chromosomes whose DNA content has been accurately determined. The size in megabases of each chromosome in the test species is calculated in relation to the control chromosomes. The sum of the individual measurements equals the genome size of the species, and this estimate correlates well with genome sizes determined by sequencing, at least for species in which the draft DNA sequence is believed to be complete. The results have been used to correct many errors in the genome size database in which genome sizes have been estimated by less precise methods [97].

Several other molecular methods have been introduced to identify chromosome deletions and duplications at high resolution. Comparative genome hybridization (CGH) depends on the comparison of the patients genomic DNA with that of a normal control [98]. In essence it is a form of reverse chromosome painting in which the test DNA labelled in one colour is mixed with genomic DNA from a normal control labelled in another colour. The mixture is hybridized to normal metaphases and the ratio of the two colours is determined by scanning along each chromosome. A predominance of the subject DNA colour indicates a duplication, while a predominance of the control DNA colour indicates a deletion. Array CGH is a more precise development of the method in which the mixture of test and control DNAs is hybridized to DNA spotted onto slides (chips). The spots contain DNA from marker sequences from the human genome database chosen to represent points along each chromosome. Arrays can be of low resolution, for example containing 3000 markers spaced at 1 Mb intervals, or high resolution containing over one million markers. The arrays are screened for imbalances along the lines used in chromosomal CGH. Various improvements of this technique have been introduced with the consequence that microarrays now replace much of routine diagnostic work in clinical cytogenetics as the technique has higher resolution, can be automated and is less time consuming than conventional karyotyping. Cytogenetics will continue to advance by the application of new sequencing strategies and by additional innovative methods.

## Conclusion

The history of human cytogenetics has been punctuated by the introduction of new technology which on each occasion has led to the discovery of an increasing number of smaller chromosome aberrations associated with disease. Modern molecular methods are capable now of identifying chromosome aberrations at the level of the DNA sequence. One of the problems of this refinement is the difficulty in distinguishing between pathological events and normal copy number variation (CNV), [99] but this will resolve with increasing experience and by keeping detailed records of CNVs in normal and diseased populations. It is suggested here that now is the time to pay greater attention to the basic structure of chromosomes, particularly the chromosomal proteins, for there is still so much more to be discovered for application in molecular cytogenetics.
